# A cognitive resilience gene expression signature in excitatory intratelencephalic cortical neurons

**DOI:** 10.1002/alz.095063

**Published:** 2025-01-09

**Authors:** Lauren A Fish, Maria Telpoukhovskaia, Jonathan Algoo, Niran Hadad, Brianna Gurdon, Miko Dai, Andrew R Ouellette, Sarah M Neuner, Amy R Dunn, Jon A. L. Willcox, Yiyang Wu, Logan C. Dumitrescu, Orhan Bellur, Jigang Zhang, Kristen MS O'Connell, Eric B. Dammer, Nicholas T Seyfried, Sukalp Muzumdar, Jesse Gillis, Paul Robson, Matthias Arnold, Timothy J Hohman, Vivek M. Philip, Vilas Menon, Catherine C. Kaczorowski

**Affiliations:** ^1^ University of Michigan, Ann Arbor, MI USA; ^2^ The Jackson Laboratory, Bar Harbor, ME USA; ^3^ Columbia University, New York, NY USA; ^4^ The University of Maine, Orono, ME USA; ^5^ University of Maine Graduate School of Biomedical Science and Engineering, Orono, ME USA; ^6^ Icahn School of Medicine at Mount Sinai, New York, NY USA; ^7^ Vanderbilt Memory and Alzheimer’s Center, Vanderbilt University Medical Center, Nashville, TN USA; ^8^ Institute of Computational Biology, Helmholtz Zentrum München, German Research Center for Environmental Health, Neuherberg Germany; ^9^ Emory University School of Medicine, Atlanta, GA USA; ^10^ Cold Spring Harbor, Cold Spring Harbor, NY USA; ^11^ University of Toronto, Toronto, ON Canada; ^12^ University of Connecticut, Farmington, CT USA; ^13^ The Jackson Laboratory for Genomic Medicine, Farmington, CT USA; ^14^ Duke University, Durham, NC USA; ^15^ Institute of Computational Biology, Helmholtz Zentrum München, German Research Center for Environmental Health, Neuherberg, Bavaria Germany; ^16^ Vanderbilt Memory and Alzheimer's Center, Vanderbilt University Medical Center, Nashville, TN USA; ^17^ Center for Translational & Computational Neuroimmunology, Department of Neurology, Columbia University Irving Medical Center, New York, NY USA

## Abstract

**Background:**

Alzheimer’s disease (AD) is a devastating form of dementia, and its prevalence is rising as human lifespan increases. Our lab created the AD‐BXD mouse model, which expresses AD mutations across a genetically diverse reference panel (BXD), to identify factors that confer resilience to cognitive decline in AD. This model mimics key characteristics of human AD including variation in age of onset and severity of cognitive decline.

**Method:**

To facilitate discovery of conserved mechanisms of resilience to AD, we generated a cross‐species single‐nuclei transcriptomic dataset from normal and AD human (ROSMAP) and AD‐BXD mouse frontal cortex tissue. We interrogated resilience‐associated gene expression signatures, validated resilience candidate genes with human reference data, and used a druggability ranking and drug repositioning pipeline to nominate drugs to promote resilience to AD. To learn more about the context of resilience gene expression, we used a hierarchical mapping algorithm to predict anatomical locations of cells expressing resilience gene signatures.

**Result:**

We found the strongest gene expression signature associated with cognitive resilience to AD arises from excitatory layer 4/5 (eL4/5) cortical intratelencephalic neurons. This resilience signature includes genes involved in synaptic plasticity, vesicle transport, and axonal and dendritic development. We found that 27 of the 61 genes in the resilience signature are druggable and identified several candidate drugs for further investigation (Telpoukhovskaia et al., 2022). We also identified genes expressed across a continuum of cognitive performance. Our hierarchical mapping approach showed that the eL4/5 neurons expressing resilience signature genes are distributed throughout the frontal cortex, mainly in the somatomotor area.

**Conclusion:**

We identified 61 candidate resilience genes to target with new or existing drugs. We also determined that expression of resilience candidate genes occurs in eL4/5 neurons in the somatomotor region of the cortex. Ongoing projects in the lab aim to evaluate efficacy of nominated drugs and profile learning‐specific proteomes of eL4/5 neurons in resilient and susceptible AD‐BXD strains. When integrated with existing genetic, behavioral, and pathological data, our work will elucidate the cellular, molecular, and genetic mechanisms that contribute to cognitive resilience in face of neurodegenerative disease pathology.

